# Are Changes in Physical Activity, Pain, and Quality of Life in Patients with Knee Osteoarthritis After Exercise Therapy and Education Beyond Normal Fluctuations? A Comparative Study

**DOI:** 10.3390/jcm14103406

**Published:** 2025-05-13

**Authors:** Mahdie Rafiei, Supratim Das, Ewa M. Roos, Søren T. Skou, Jan Baumbach, Linda Baumbach

**Affiliations:** 1 Institute for Computational Systems Biology, University of Hamburg, 22761 Hamburg, Germany; 2 Department of Health Economics and Health Services Research, University Medical Center Hamburg-Eppendorf, 20246 Hamburg, Germany; linda.baumbach@uni-hamburg.de; 3 Center for Muscle and Joint Health, Department of Sports Science and Clinical Biomechanics, University of Southern Denmark, 5230 Odense, Denmark; 4 The Research and Implementation Unit PROgrez, Department of Physiotherapy and Occupational Therapy, Næstved-Slagelse-Ringsted Hospitals, 4200 Slagelse, Denmark; 5 Computational Biomedicine Laboratory, Department of Mathematics and Computer Science, University of Southern Denmark, 5230 Odense, Denmark; 6 Chair of Genome Informatics, Center for Bioinformatics, University of Hamburg, 22761 Hamburg, Germany

**Keywords:** knee osteoarthritis, exercise therapy, physical activity, pain intensity, quality of life

## Abstract

**Objective**: This study evaluates whether one-year changes in physical activity (PA), pain intensity, and quality of life (QOL) after exercise therapy and education are larger than normal fluctuations over time in individuals with knee osteoarthritis. **Method**: Patients with knee osteoarthritis participating in the Good Life with Osteoarthritis in Denmark (GLA:D^®^) exercise therapy and education program (n = 7603) and participants from the Osteoarthritis Initiative (OAI) who received no specific treatment (n = 1156) were included. PA was measured using the UCLA PA scale (1–10) in the GLA:D^®^ group and the PASE (0–531) in the OAI group. PASE scores were mapped to the UCLA distribution. Pain intensity was measured using a standardized visual analog scale (VAS, 0–100), and QOL was assessed via the KOOS QOL scale (0–100). Changes were categorized as increased, maintained, and decreased. To ensure comparability between GLA:D^®^ and OAI participants, we used entropy balancing, considering the covariables age, gender, BMI, depression, employment status, and our outcome variables at baseline. **Results**: At one year, 41% of GLA:D^®^ participants showed increased PA compared to 38% in the balanced OAI group (*p* = 0.015). Pain intensity decreased by ≥20 mm on the VAS in 39% of GLA:D^®^ and 27% of OAI participants (*p* < 0.001). QOL improved by ≥ 10 mm on the KOOS scale in 48% of GLA:D^®^ and 40% of OAI participants (*p* < 0.001). Additionally, for PA, pain, and QOL, 6%, 13%, and 7% more patients in the control group experienced worsening in these outcomes, respectively. **Conclusions**: Twelve percent more participants experienced clinically relevant pain reductions in the GLA:D^®^ group compared to OAI participants, and 3% and 8% more reported improvements in PA and QOL, respectively. Additionally, more patients in the control group experienced worsening in these outcomes. These differences indicate that clinically relevant pain improvements following exercise therapy and education may exceed normal fluctuations in patients with knee osteoarthritis.

## 1. Introduction

Insufficient physical activity (PA), defined as any bodily movement produced by skeletal muscles that requires energy expenditure [1], is a significant public health concern, associated with an increased risk of chronic diseases such as cardiovascular disease, diabetes, and osteoarthritis (OA) [2]. OA is a common, progressive joint disease characterized by joint pain, stiffness, reduced mobility, and functional limitations, most often affecting the knees and hips. These symptoms often lead to decreased PA levels and diminished quality of life (QOL) [3]. QOL refers to an individual’s perception of their position in life relative to their cultural contexts, value systems, goals, expectations, standards, and concerns [4]. PA, often provided as exercise therapy (i.e., PA designed and prescribed for specific therapeutic purposes), is beneficial for people with OA as it may reduce joint pain and stiffness, improve mobility, and enhance QOL [1]. Therefore, regular PA is recommended for the prevention and management of several chronic conditions, including OA [5]. Despite these recommendations, 13% to 60% of patients with OA still fail to meet the suggested levels of PA [6,7]. Increasing PA levels is particularly challenging, as behavioral changes require sustained effort and support [8]. Educational interventions are key to supporting this behavioral change, as they increase awareness of PA benefits, provide tools for symptom management, and empower individuals with OA to participate actively in their care [9]. Education can address misconceptions about pain and movement, improve adherence to exercise regimens, and teach self-management strategies. When combined with supervised exercise, education can lead to better adherence, improved outcomes, and greater self-efficacy [10].

Good Life with osteoArthritis in Denmark (GLA:D^®^) is an evidence-based initiative to help individuals with knee and hip OA manage their symptoms and improve their QOL [11]. The GLA:D^®^ program includes patient exercise therapy and education, which are recommended first-line treatments according to clinical guidelines [12]. Previous studies have indicated that the GLA:D^®^ program is associated with better outcomes one year after the program compared to before the program [11]. Additionally, it is suggested that the program may help increase PA levels among participants [13]. However, since GLA:D^®^ is an uncontrolled cohort collected in clinical practice, it remains unclear whether the suggested changes in PA, pain, and QOL result from the program or are merely due to natural fluctuations over time.

One of the primary challenges in observational studies is the absence of a control group, making it difficult to distinguish the true effect of an intervention from normal fluctuations in outcomes over time. While randomized controlled trial (RCTs) provide strong evidence of efficacy, their highly controlled conditions—such as strict eligibility criteria, intensive follow-up, and high adherence rates—may not fully reflect real-world patient populations. RCTs are considered the gold standard for evaluating intervention effectiveness due to their ability to minimize bias through random allocation and controlled conditions [14]. For example, multiple RCTs have demonstrated that structured exercise and education programs, such as a 12-week program for hip and knee OA, significantly improve pain, function, and well-being [15]. However, observational studies suggest that while exercise therapy is beneficial, the magnitude of improvements may be smaller than those observed in RCTs (KOOS_4_ improvement: 16 points in an RCT [16] vs. 8 points in the GLA:D^®^ Annual Report 2021 [17]). This raises an important question: Do RCTs overestimate the benefits due to controlled conditions, or do real-world variations reduce the effects? Without a proper control group in observational studies, it remains challenging to assess accurately whether changes in outcomes exceed normal fluctuations.

This research aims to address this knowledge gap by comparing changes in PA, pain intensity, and QOL at one year between participants of the GLA:D^®^ program and a matched cohort from the Osteoarthritis Initiative (OAI), who did not receive any specific treatment. Thus, we seek to determine if the change associated with the GLA:D^®^ program is above the level of normal fluctuation.

Our findings will provide valuable insights into whether the changes observed in the GLA:D^®^ program surpass normal fluctuations and contribute to the broader understanding of how structured interventions can impact PA and overall health outcomes in individuals with OA.

## 2. Method

### 2.1. Source of Data and Participants

This study is a comparative cohort study using two pre-existing datasets. Data for this study were obtained from the GLA:D^®^ (Good Life with osteoArthritis in Odense, Denmark) program and the Osteoarthritis Initiative (OAI) [11,18].

In this study, participants from the GLA:D^®^ dataset were considered the treatment group, as they received structured education and supervised exercise therapy. GLA:D^®^ is a standardized, structured exercise and educational program in Denmark for patients suffering from hip and knee OA to improve their outcomes. It is designed to improve outcomes in people with OA. The program includes two to three educational sessions on OA symptoms, pain mechanisms, management, and self-care; twelve 60 min supervised neuromuscular exercise therapy sessions over six weeks (twice weekly); and individual physiotherapy consultations at the start and end of the program. The program is delivered by physiotherapists who have completed a two-day training course, and data are collected through a national registry at the baseline, after the program, and at a 12-month follow-up. The registry also includes clinician- and patient-reported outcomes, functional tests, and treatment setting information. Detailed descriptions of its educational components, neuromuscular exercise protocol, and overall framework have been published previously [11,19] and are available in our supplementary analysis. In the GLA:D^®^ program, OA is clinically diagnosed based on symptoms such as joint pain, stiffness, and functional limitations, without requiring radiographic confirmation as recommended in international guidelines [11,20].

GLA:D was established in 2013 and is regularly updated to include new evidence. Our inclusion criteria for patients were that they stated the knee as their primary joint of complaint, provided data between 2014 and 2022, and had complete data on PA, pain, and QOL at both the baseline and 12-month follow-up.

The OAI dataset, of patients with OA, functions as the control group, since this group did not receive any specific treatment but care as usual. The OAI is a multicenter, ten-year observational study of men and women, designed to identify risk factors and biomarkers for the development and progression of knee OA. It thus includes patients at risk and with OA. It is sponsored by the National Institutes of Health (NIH) [18]. The OAI was launched in 2002 in the United States and completed in 2015, focusing on knee OA. The primary goal of the OAI is to develop a public-domain research resource to facilitate the scientific evaluation of biomarkers for OA as potential surrogate endpoints for disease onset and progression. The OAI collected a wide range of data, including clinical assessments (e.g., knee symptoms and function), imaging (X-rays and MRIs of knees and other joints), and biospecimens (blood, urine, DNA, and lymphocytes). Baseline visits for the OAI took place between February 2004 and May 2006, with each participant’s 12-month follow-up occurring approximately one year after their baseline visit, for a total of 12 follow-ups. In the OAI dataset, knee OA is defined using both radiographic criteria (Kellgren–Lawrence grade ≥2) and the presence of frequent knee pain over the past 12 months [18]. We included only participants with knee OA and complete data on PA, pain, and QOL at both the baseline and 12-month follow-up.

Ethical approval was not required for the GLA:D^®^ registry, according to the North Denmark Region ethics committee. The OAI dataset is publicly available and IRB-approved [16].

### 2.2. Outcome

We calculated the observed changes in PA, pain intensity, and QOL from the baseline to after one year in both datasets.

**Figure 1:**
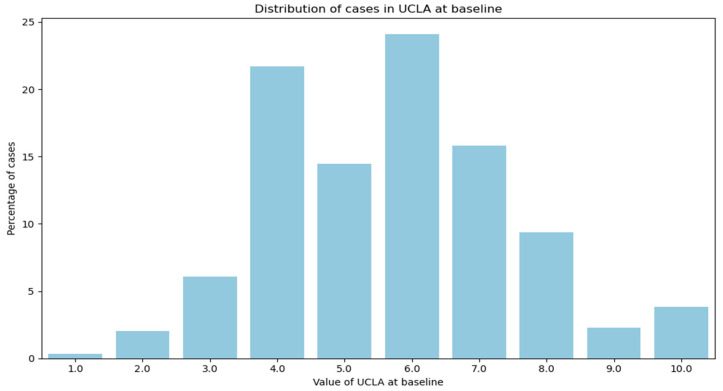
Distribution of UCLA scores at the baseline in the GLA:D^®^ data.

**Figure 2:**
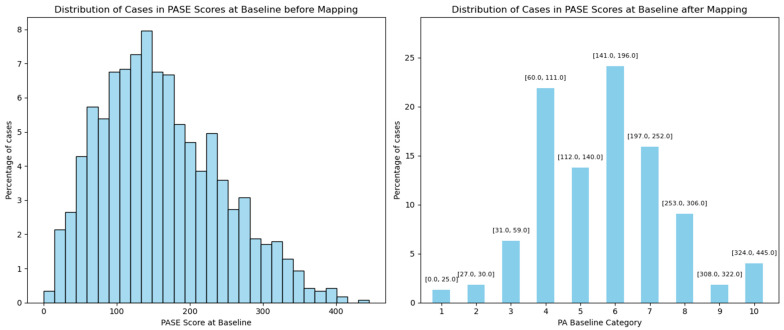
Distribution of PASE scores at the baseline before and after mapping. The left panel shows the distribution of the baseline PA Scale for the Elderly (PASE) scores in the OAI dataset. The right panel shows the number of cases within the cut points of the baseline PASE scores after mapping based on the UCLA distribution in the GLA:D^®^ dataset.

In the GLA:D^®^ dataset, PA was assessed using the UCLA scale, where participants responded to the question, “What is your current activity level during the last 4 weeks?” with ratings on a scale from 1 (low) to 10 (high). In the OAI dataset, PA was measured using the PA Scale for the Elderly (PASE), which assesses activities in three main domains: leisure, household, and occupational. Scores range from 0 to 531, with higher scores indicating greater PA levels. Due to differences in the PA scales between the GLA:D^®^ and OAI datasets, the PASE scores from the OAI were mapped to the UCLA scale (Figure 1 and Figure 2). This was performed by dividing the PASE scores into 10 parts based on the distribution of UCLA scores in the GLA:D^®^ dataset. This facilitated a direct and granular comparison of PA between the two datasets.

Pain intensity in the GLA:D^®^ dataset was determined using a visual analog scale (VAS), a validated and widely used measure of pain in patients with OA. The VAS has demonstrated good reliability, validity, and responsiveness in this population [21,22]. The question asked to the patients was “I would like you to think about a scale that goes from no pain (0) to worst pain imaginable (100) that best represents your knee pain during the last week (before June 2020, it was asked for the “last month”)”. Participants indicated their pain level on a VAS ranging from 0 to 100 mm, where 0 represented “no pain” and 100 signified “worst possible pain”.

In the OAI dataset, pain intensity was measured by the severity of knee pain over the past 7 days, rated on a scale from 0 to 10. This format corresponds to the Numeric Pain Rating Scale (NPRS), a validated and widely used measure with strong reliability, validity, and responsiveness in OA populations [21,23]. For comparison with the GLA:D^®^ dataset, these OAI pain scores were converted to a 0 to 100 scale, aligning with the VAS used in the GLA:D^®^ dataset. This conversion enabled a consistent evaluation of pain intensity changes across both datasets.

QOL was assessed using the Knee Injury and Osteoarthritis Outcome Score (KOOS) QOL subscale in both the GLA:D^®^ and OAI datasets. This subscale ranges from 0 to 100, with higher scores indicating better QOL. The KOOS QOL subscale has demonstrated good reliability, validity, and responsiveness in individuals with knee OA [24]. Since both datasets used the same KOOS QOL subscale, the measures were directly comparable without further mapping.

### 2.3. Covariables

To ensure comparability between the GLA:D^®^ and OAI datasets, we first identified and matched relevant variables that were comparable across both datasets. Based on the literature, highlighting factors influencing PA, pain intensity, and QOL, we decided to include age, gender, BMI, depression, and employment status as covariables [25,26,27]. These factors were consistently available in both datasets and are known to influence PA, pain intensity, and QOL. Including these variables allows us to account for potential primary differences between our treatment and control groups.

Table 1 details the necessary translations and changes for each variable across the two datasets. The variables age, gender, BMI, depression, and baseline QOL, did not require any changes as they were directly comparable between the two datasets.

Employment status in the GLA:D^®^ dataset was categorized as (1) working for pay, (2) not working in part due to health, and (3) not working for other reasons. In the OAI dataset, we categorized employment status as (1) employed/student, (2) on sick leave full-time, and (3) unemployed.

Baseline pain in the OAI dataset required a translation to align with the GLA:D^®^ dataset. The OAI dataset measured pain separately for the left and right knees. For each participant, we compared the pain levels between the left and right knees and chose the higher reported pain levels as a representative measure of knee pain. This approach allowed us to translate the OAI pain measurements into a format comparable to the visual analog scale (VAS) used in the GLA:D^®^ dataset.

Baseline PA levels in the OAI were translated based on the baseline PA UCLA scale in the GLA:D^®^, as described above.

### 2.4. Matching of Participants

For our analyses, we matched the participants between the GLA:D^®^ and the OAI datasets based on the covariables mentioned above. This step was critical in mitigating any potential bias arising from differing patient characteristics and variable definitions. We employed entropy balancing, a reweighting technique designed to create a balanced dataset by adjusting the weights of the control group to match the covariable moments of the treatment group [28]. Entropy balancing ensures that the weighted control group has the same distribution of covariables as the treatment group. This method involves the following steps:

1. Computing covariable means: The process begins by calculating the covariable means in the treatment group.
2. Optimizing control group weights: Next, the weights of the control group are optimized to minimize the differences between the weighted means of the control group’s covariable and the treatment group’s covariable means.
3. Creating a balanced dataset: Finally, control samples are replicated according to these optimized weights to create a balanced dataset that closely resembles the treatment group.

By applying entropy balancing to the OAI dataset, we created a balanced control group comparable to the GLA:D^®^ treatment group, facilitating a more accurate estimation of treatment effects. This process reduced bias and improved the validity of the comparative analysis [28,29,30].

### 2.5. Statistical Analysis Methods

After the initial mapping of outcomes and covariables, as described and illustrated in Figure 1 and Figure 2, we calculated the changes in outcomes from the baseline to the one-year follow-up. These changes were categorized separately for PA, pain intensity, and QOL.

For PA, the GLA:D^®^ dataset used the UCLA scores to assess changes in PA, which we classified into increases, maintained levels, or decreases. Similarly, the OAI dataset used the created UCLA PA scores to categorize PA changes as increases, maintained levels, or decreases.

For pain intensity, the analysis was based on changes of at least 20 mm on the VAS, which is considered clinically significant [31]. In both the GLA:D^®^ and OAI datasets, pain intensity changes were categorized as an “increase” or “decrease” if a change of at least 20 mm occurred; otherwise, “maintained” was coded [32,33].

For QOL, both the GLA:D^®^ and OAI datasets analyzed the changes from the baseline, with a threshold of 10 points on the KOOS scale, which is considered a clinically significant difference [20]. Changes in QOL were categorized in both datasets as an increase or decrease of at least 10 points on the KOOS scale. If no change or a change of fewer than 10 points occurred, maintained was coded as the outcome.

This approach ensured that each outcome—PA, pain intensity, and QOL—was evaluated similarly within the GLA:D^®^ and OAI datasets, using predefined thresholds for clinical significance for pain intensity and QOL.

Initially, we analyzed without applying entropy balancing (control group) to observe the raw differences between the datasets. Subsequently, we applied entropy balancing (balanced control group) to match individuals from the OAI to those of the GLA:D^®^ data based on the identified covariable. This reweighting technique ensured that the distributions of covariables in the control group matched those in the treatment group, allowing for a more accurate comparative analysis.

We utilized the chi-squared test to compare differences in the distribution of categorical outcomes (increased, maintained, and decreased) between the GLA:D^®^ and OAI datasets. This statistical test assessed whether there were significant differences in the observed frequencies of these outcomes between the treatment and control groups.

All data analyses were performed in Python version 3.11 using Scikit-learn, Pandas, and NumPy libraries, ensuring robust and reproducible analyses.

### 2.6. Supplementary Analyses

To secure the validity of our comparison in changes in PA between the GLA:D^®^ and OAI datasets, we performed an additional analysis utilizing a different approach to map the PASE scores of the OAI to the UCLA PA score from GLA:D^®^. Therefore, we categorized the PA scoring scales into low, moderate, and high PA as defined by the literature. UCLA scores were categorized into 1–4 (low), 5–6 (moderate), and 7–10 (high), as suggested by relevant literature [32]. Similarly, PASE scores were divided into Tertile 1 (low), ranging from 0 to 152, Tertile 2 (moderate), from 153 to 207, and Tertile 3 (high), from 208 to 445, based on established cut points in the literature [33]. This categorization allowed for a standardized comparison of PA levels between the two datasets, facilitating a more meaningful analysis of the impact of the treatment programs on PA, pain intensity, and QOL. The remaining steps of the analyses remained the same.

To assess the impact of pain medication use on our study outcomes, we included it as an additional covariable in a second supplementary analysis. Pain medication use was measured differently in the two datasets. In the GLA:D^®^ dataset, participants were asked at the baseline, “Does the patient take any pain medications, including herbal or dietary supplements in the last 2 weeks?”. In contrast, the OAI dataset assessed medication use by asking, “Used nonprescription or prescription NSAIDS (e.g., Aspirin, Ibuprofen…) for joint pain or arthritis more than half the days of the month, past 30 days”. After incorporating this variable, we re-evaluated changes in PA, pain intensity, and QOL across the treatment and control groups. This analysis allowed us to examine whether pain medication use influenced the observed effects of the intervention.

## 3. Result

### 3.1. Participants

**Figure 3:**
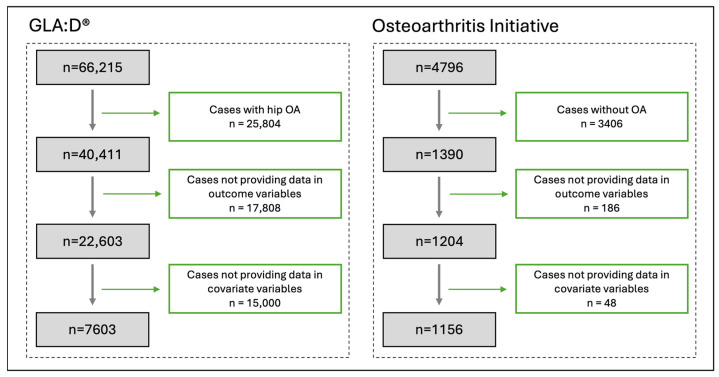
Flow chart of the included patients.

Our GLA:D^®^ dataset included 66,215 patients, and after excluding patients who did not match our inclusion criteria (Figure 3), 7603 patients remained for analysis. In the OAI dataset, 4796 patients were enrolled at the beginning of the study, and after excluding those not related to our study, 1156 patients were included. For detailed inclusion steps, see Figure 3.

### 3.2. Patient Characteristics

Table 2 provides the characteristics of the participants before and after utilizing entropy balancing, including means and standard deviations for continuous variables.

To compare age characteristics across groups, we observed that the GLA:D^®^ participants had a wider age range (23–85 years, range = 62) than the OAI participants (45–79 years, range = 34). A two-sample t-test confirmed that this difference was statistically significant (t = −16.04, *p* < 0.001). Age was therefore included as a covariate in the entropy balancing to adjust for baseline differences between groups.

Additionally, we would like to highlight that among the 7603 GLA:D^®^ participants, 10% (n = 762) reported a history of joint replacement in a hip or knee (missing: n = 52), 36% (n = 2721) had previously consulted a physiotherapist for current joint problems, and 74% (n = 5646) reported taking pain medications at the baseline. At the 12-month follow-up, 5% (n = 364) reported a knee or hip replacement since the start of GLA:D^®^ (missing = 2790). Unfortunately, we lack information on additionally received physiotherapeutic care in the GLA:D^®^ data, as not all of our 7603 cases responded to the question, ‘Since starting up GLA:D^®^, have you consulted a physiotherapist because of hip/knee problems (other than related to GLA:D^®^)?’ in the GLA:D^®^ dataset. Additionally, 27% (n = 2079) reported taking pain medications (missing = 2971).

In contrast, among the 1156 OAI participants, at the baseline, only 1% (n = 16) had a history of left or right knee replacement surgery (missing: n = 98), and 6% (n = 65) had seen a chiropractic care practitioner for arthritis or joint pain (missing: n = 1045). Finally, 32% (n = 368) reported using pain medications (missing = 2). At the first 12-month follow-up, still, only 1% (n = 3) of OAI participants had a history of left or right knee replacement surgery (missing: n = 1053), and 5% (n = 60) reported visiting a chiropractic care practitioner for arthritis or joint pain (missing: n = 1064). Additionally, 22% (n = 250) reported taking pain medications (missing: n = 255). Supplementary Table S1 presents the detailed questions and characteristics of our additional variables regarding received treatments.

### 3.3. Changes in PA, Pain Intensity, and QOL

The analysis examined changes in PA, pain intensity, and QOL from the baseline to the one-year follow-up, categorized as increases, maintained levels, or decreases.

1. Physical Activity

**Figure 4:**
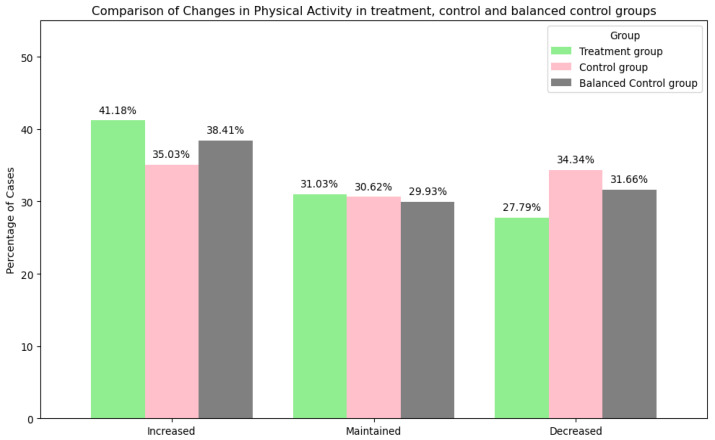
Comparison of changes in PA from baseline to follow-up in treatment, control, and balanced control groups. The bar chart illustrates the percentage of cases that experienced changes in PA. The categories include ‘increased’, ‘maintained’, and ‘decreased’ activity levels, comparing treatment, control, and balanced control groups. Group differences were statistically significant (χ^2^(2) = 8.93, *p* = 0.0115).

The comparison of changes in PA between the treatment group (GLA:D^®^), control group (OAI), and balanced control group (OAI participants after entropy balancing) is illustrated in Figure 4. The treatment group showed a higher percentage of increased PA (41%) compared to the control group (35%) and balanced control group (38%). Also, the control group showed a higher percentage of decreased PA (34%) compared to the treatment (28%) and balanced control groups (32%).

The chi-squared test for the PA change category yielded a chi-squared value of 8.93, with a *p*-value of 0.0115 and 2 degrees of freedom. This result indicates a statistically significant difference in PA changes between the groups.

2. Pain Intensity

A change in pain intensity was considered if a difference of more than 20 mm on the VAS occurred. A higher percentage of participants in the treatment group experienced such a significant decrease in pain intensity (39%) compared to the control group (29%) and balanced control group (27%).

**Figure 5:**
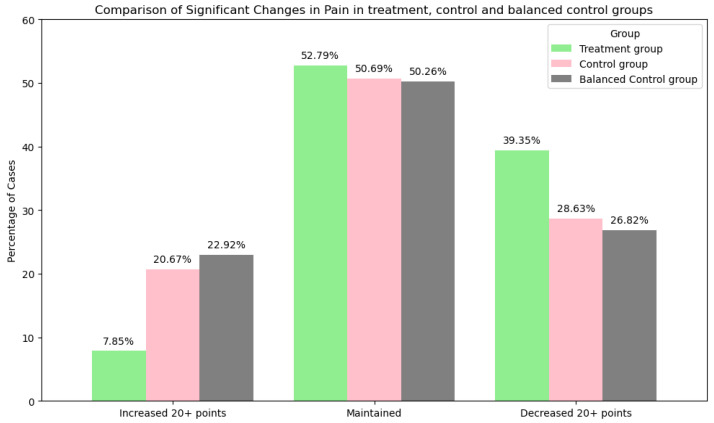
Comparison of significant changes in pain from the baseline to the follow-up in the treatment, control, and balanced control groups. This figure shows the percentage of cases with significant improvements in (20+ mm on the VAS), decreases in (20+ mm on the VAS), or maintained pain intensity. Group differences were statistically significant (χ^2^(2) = 258.21, *p* < 0.001).

The treatment group had a lower percentage of participants with a significant increase in pain intensity (8%) compared to the control group (21%) and balanced control group (23%). Changes in pain intensity are shown in Figure 5.

The chi-squared test for the pain change category across the treatment and balanced control groups produced a chi-squared value of 258.21, with a *p*-value of 0.001 and 2 degrees of freedom. This result indicates a significant difference in pain intensity changes between the groups.

3. Quality of Life

**Figure 6:**
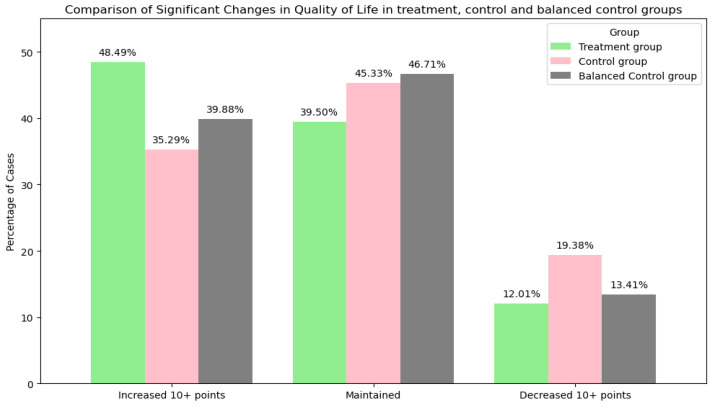
Comparison of significant changes in QOL from the baseline to the follow-up in the treatment, control, and balanced control groups. This figure shows the percentage of cases with significant improvements in (10+ mm on the VAS), decreases in (10+ mm on the VAS), or maintained QOL scores. Group differences were statistically significant (χ^2^(2) = 33.03, *p* < 0.001).

A change in QOL was considered to be > 10 mm on the KOOS scale in either direction. The treatment group had a higher percentage of participants with a significant increase in QOL (48%) compared to the control group (35%) and the balanced control group (40%). Also, the control groups showed a higher percentage of participants with decreased QOL (19% and 13%) compared to the treatment group (12%). Changes in QOL are shown in Figure 6.

The chi-squared test for the QOL change category across treatment and balanced control groups resulted in a chi-squared value of 33.03, with a *p*-value of 0.001 and 2 degrees of freedom. The test reveals a significant change in QOL distribution between the groups.

### 3.4. Supplementary Analyses

The supplementary analysis, based on established PA cut points from the literature [32,33], is presented in the supplementary analysis file. Overall, the findings were similar to our main findings. In the treatment group, 27.52% and 17.99% in the balanced control group increased their PA. These findings also reveal a statistically significant difference in PA changes between the treatment and balanced control groups.

The result of the pain medication as an additional covariable is detailed in the supplementary analysis. Incorporating this variable led to a slight change in OAI cases, decreasing from 1156 to 1154 due to missing values. The treatment group showed the highest improvements, with 39.35% experiencing pain reduction (≥ 20 points), 48.49% reporting increased QOL (≥ 10 points), and 41.18% increasing PA. In comparison, the control and balanced control groups had lower percentages for pain reduction (28.68% and 26.86%), QOL improvement (35.36% and 39.69%), and PA increase (35.01% and 38.30%), respectively.

## 4. Discussion

### 4.1. Summary

Our study aimed to evaluate whether the changes associated with the GLA:D^®^ program in PA, pain intensity, and QOL surpass normal fluctuations by comparing participants of the program to a matched cohort from the OAI, who did not receive specific treatment. Using entropy balancing, we ensured comparability between groups. At one year, significantly more GLA:D^®^ participants improved PA (41% vs. 38%), reported reduced pain intensity (39% vs. 27%), and increased QOL (48% vs. 40%) compared to the OAI group (*p* < 0.05). Chi-squared tests confirmed that these differences were statistically significant, suggesting that the program’s benefits extend beyond natural variation. However, while PA changes were statistically significant, their clinical relevance remains uncertain. Notably, slightly more OAI participants (6%, 13%, and 7%) reported worsening in PA, pain intensity, and QOL, reinforcing the positive impact of exercise and education.

### 4.2. Methodological Considerations

To the best of our knowledge, this is the first study to assess the GLA:D^®^ program’s effectiveness in a real-world setting using a matched observational design, ensuring comparability with a non-intervention cohort. This methodology might serve as an inspiration for future research, as it strengthens the evidence on the efficacy of a treatment in a real-world setting. While RCTs remain the gold standard for evaluating interventions, they may not fully reflect real-world settings. Our study, in contrast, used an observational approach, making it necessary to apply entropy balancing to account for baseline differences between self-selected GLA:D^®^ participants and untreated OAI participants. While RCTs eliminate bias through randomization [34], our approach adjusts for differences statistically, enabling a comparison in the real-world context. Alternative methods such as propensity score matching [30] or target trial [35] emulation could have also been applied to address confounding and strengthen causal inference. However, we selected entropy balancing due to its ability to exactly balance covariates and retain the full sample, minimizing the loss of data while improving comparability between groups.

A factor to consider when interpreting our findings is our mapping method for evaluating PA. To ensure comparability, we mapped PASE scores from the OAI group to the UCLA scale and validated this approach through supplementary analyses. The results of these additional analysis with a categorization of PA into low, moderate, and high strengthened the robustness of our results. The results confirm that the observed differences in PA changes between the groups are not due to scale discrepancies but reflect true differences.

Further, it is important to discuss the characteristics of enrollment and how they might affect our findings. The GLA:D^®^ data were collected from a Danish population, while the OAI data originated from the United States. Cultural, healthcare, and socioeconomic differences between these populations may have contributed to differences in changes in outcomes. Furthermore, participants of the GLA:D^®^ and OAI groups may exhibit differences due to the enrollment process. GLA:D^®^ enrolls patients seeking structured exercise therapy and education in a clinical setting, while the OAI passively recruits participants for a long-term observational study without assigning them to a specific treatment. This difference means GLA:D^®^ participants are actively engaged in symptom management, whereas OAI participants may currently not be looking for healthcare. However, we accounted for potential differences in the characteristics between datasets, including baseline pain, by applying entropy balancing, which is one of the study’s strengths. Entropy balancing is a robust statistical method that controls for differences at the baseline. The approach allowed us to create a comparable sample for the GLA:D^®^ participants in the OAI dataset, enhancing the validity of our findings.

While key participant characteristics such as age, gender, BMI, depression, employment status, baseline PA, pain intensity, and QOL were included in the balancing, some potentially influential covariables—such as previously received treatments, smoking habits, sitting time, and educational status—were not incorporated due to missing data or difficulty in harmonizing across datasets, and were consequently not included in the balancing process [26,36,37,38].

### 4.3. Interpretation of Results

An important consideration in our findings is regression to the mean (RTM), where individuals with extreme baseline values naturally show improvements over time, even without intervention. A study by Englund and Turkiewicz [39], using OAI data, found that RTM can lead to small improvements in physical function and QOL. However, RTM alone cannot explain the greater improvements in the GLA:D^®^ group, particularly in pain intensity, where significantly more participants experienced a ≥ 20 mm VAS pain reduction. Including a control group and using entropy balancing minimized RTM biases, suggesting that the observed improvements in PA, pain, and QOL are clinically meaningful and not just statistical fluctuations.

Although fewer GLA:D^®^ participants reported a decrease in QOL compared to the control groups, it is important to acknowledge that some experienced worse QOL. This is likely due to the progressive nature of OA, which leads to measurable structural and symptomatic decline over time in 12–29% of the patients [40]. An alternative explanation could be that these patients did not maintain an increase in their PA and therefore experienced worsening over time. However, our findings show that a greater proportion of participants in the GLA:D^®^ group experienced improvements, suggesting that a structured intervention might change the patient’s lifestyle and can prevent worsening symptoms.

While OAI participants did not receive a standardized intervention like GLA:D^®^, some may have undergone and received treatments such as exercise therapy, other physiotherapy, or surgery, which could have influenced the results. However, since these treatments were not included in our analysis due to high missing values, their impact remains unclear. Below, we discuss the potential impact of pain medication, joint replacement surgery, and consultations with physiotherapy.

The supplementary analysis showed that incorporating pain medication slightly altered case distribution but did not significantly affect treatment effectiveness. Previous studies also suggest that pain medication use is weakly associated with outcomes in OA patients receiving education and exercise programs [41]. Additionally, a recent RCT found that patients who initially relied on analgesics benefited more from structured exercise therapy, suggesting that GLA:D^®^ may not only provide pain relief to patients but also help reduce their dependence on medication [42,43]. While pharmacological treatment can provide short-term relief of OA symptoms, its effect on long-term improvements in QOL and PA is limited [44,45]. In our supplementary analysis, the inclusion of pain medication use as a covariate did not substantially change the outcomes. This supports the notion that the improvements observed in the GLA:D^®^ group may be attributable to the structured exercise and education intervention. Furthermore, to account for relevant comorbidities, we included depression as a covariate in our entropy balancing. Depression is known to influence pain perception, physical function, and QOL, and its inclusion supports our findings.

While knee replacement surgery is generally associated with improved outcomes [46,47,48], studies indicate that approximately 5% to 10% of patients may experience the surgery and related care as “treatment failure” in any disease [49,50,51,52,53]. In particular, a study of knee replacement [54] report that approximately 20% of patients undergoing knee replacement experience an unfavorable pain outcome, indicating persistent or insufficient pain relief following the procedure. In our study, 10% of GLA:D^®^ participants had a joint replacement at the baseline (not necessarily the index joint), and 1% of them underwent another knee or hip replacement since starting the program. Due to 90% missing data for this outcome in the OAI control group, we could not respect its direct impact in our analyses. However, evidence suggests that prior knee surgeries do not substantially affect the benefits of exercise therapy. Grønne et al. [55] found that patients with previous knee surgeries—including procedures other than total knee replacement—improved similarly in pain, QOL, and walking speed after GLA:D^®^, supporting the notion that prior surgery does not necessarily hinder the effectiveness of structured exercise therapy.

The OAI cohort may have included individuals who received physiotherapy or other structured exercise interventions outside the study context, potentially influencing the observed differences. However, the lack of similar information on received physiotherapy and other exercise programs prevented us from controlling for its impact on entropy balancing, leaving uncertainty about the role of additional treatments in both groups. Future studies should aim to collect more detailed data on supervised exercise participation.

### 4.4. Limitations

While this study provides valuable insights into the effectiveness of the GLA:D^®^ program, some limitations should be considered.

The OAI control group was smaller than the GLA:D^®^ group due to the exclusion of non-OA patients. While entropy balancing ensured comparability between groups, it does not make the OAI control group fully representative of all untreated OA patients. The smaller control group reduces statistical power, increasing variability and making the results more sensitive to individual differences [56]. Future studies should validate these findings using larger, more diverse control groups to improve generalizability.

Furthermore, a significant proportion of missing values in the GLA:D^®^ dataset (n = 17,808 cases) raise concerns about selection bias, particularly if those who did not provide data systematically differ from those who did. If patients lost to the follow-up had different baseline characteristics or outcomes compared to those retained in the study, this could distort the observed effects of the GLA:D^®^ program. For instance, if participants who experienced less improvement or worsened symptoms were more likely to drop out, our study may overestimate the program’s effectiveness. Registry-based studies often face these challenges, and without comparison, potential bias remains uncertain. However, from previous studies utilizing the GLA:D^®^ data, we know that in the comparison of patients lost to the follow-up and patients with missing values included in the analysis, the differences in characteristics remained not clinically relevant [57]. Future studies could, though, assess missing cases, apply imputation, and include key predictors to improve reliability.

Additionally, the severity of OA could not be directly compared between the two datasets, since GLA:D^®^ uses a clinical diagnosis based on symptoms, whereas the OAI uses radiographic criteria. Although this prevents a direct evaluation of disease stage equivalence, we included baseline pain intensity, a proxy for symptom severity, as a covariate. It mitigated the impact of OA severity across the treatment and control groups.

## 5. Conclusions

After one year, 12% more GLA:D^®^ participants experienced pain reduction compared to OAI participants, while 3% and 8% more reported improvements in PA and QOL, respectively. Furthermore, more participants in the OAI group experienced a worsening in these outcomes. These differences suggest that the benefits of exercise therapy and education extend beyond normal fluctuations in patients with knee OA.

## Figures and Tables

**Table 1:** Variable translation and changes across the GLA:D^®^ and OAI datasets.

GLA:D^®^			OAI	
Variable	Original Variable	Translation	Original Variable	Translation
Age	The current age in years	No change	The current age in years	No change
Gender	1: Male 2: Female	No change	1: Male 2: Female	No change
BMI		No change		No change
Depression	1: I am not anxious or depressed 2: I am slightly anxious or depressed 3: I am moderately anxious or depressed 4: I am severely anxious or depressed 5: I am extremely anxious or depressed	No change	1: Rarely/none of the time (< 1 day) 2: Some of the time (1–2 days) 3: Much of the time (3–4 days) 4: Most or all of the time (5–7 days)	No change
Employment status	1. Employed/student 2. On sick leave full-time 3. On sick leave part-time 4. Retired 5. Unemployed 6. Self-imposed early retirement 7. Early retirement due to low ability to work	1: Works for pay 2: Not working in part due to health 3: Not working other reasons	1: Works for pay 2: Unpaid work for family business 3: Not working in part due to health 4: Not working other reasons	1: Employed/student 2: On sick leave full-time 3: Unemployed
Baseline PA	Categorical variable from 1 to 10	No change	Continuous variable from 0 to 445	Mapped based on UCLA distribution
Baseline pain	0 to 100	No change	Included two variables: 1. Pain in the left knee, 0 to 10 2. Pain in the right knee, 0 to 10	1. Left knee > right knee ==> left knee Left knee < right knee ==> right knee 2. Converted from 0 to 10 to 0 to 100 based on the VAS
Baseline QOL	0 to 100	No change	0 to 100	No change

**Table 2:** Baseline characteristics and outcome changes for participants in GLA:D^®^, OAI control group, and balanced OAI control group. Depression and employment status are categorical variables. Outcome changes are reported using clinically meaningful thresholds.

Variables	GLA:D^®^ Included Cases, Mean or Number of Cases n = 7603	Control Group (OAI) Included Cases, Mean or Number of Cases n = 1156	Balanced Control Group (OAI) Included Cases, Mean or Number of Cases n = 1156
**Co-Variable**			
Age (years)	Min: 23 Max: 85 Mean: 56.69 SD: 6.81	Min: 45 Max: 79 Mean: 61.15 SD: 9.083	Min: 45 Max: 79 Mean: 56.58 SD: 7.81
Gender	1: Male: 2279 (29.97%) 2: Female: 5324 (70.03%)	1: Male: 642 (55.54%) 2: Female: 514 (44.46%)	1: Male: 414 (35.81%) 2: Female: 742 (64.19%)
BMI (kg/m^2^)	Min: 17.03 Max: 72.27 Mean: 29.59 SD: 5.82	min: 18.20 max: 48.70 mean: 30.10 SD: 4.80	min: 18.2 max: 46.7 mean: 29.61 SD: 4.87
Depression (1–4, low to high)	Rarely: 7188 (94.56%) Sometimes: 314 (4.13%) Often: 88 (1.16%) Usually: 13 (0.17%)	1: Rarely: 873 (75.52%) 2: Sometimes: 231 (19.98%) 3: Often: 31 (2.68%) 4: Usually: 21 (1.82%)	1: Rarely: 1018 (88.06%) 2: Sometimes: 134 (11.59%) 3: Often: 3 (0.26%) 4: Usually: 1 (0.09%)
Employment status	Employed/student: 6782 (89.17%) On sick leave full-time: 451 (5.93%) Unemployed: 370 (4.87%)	1: Employed/student: 715 (61.85%) 2: On sick leave full-time: 79 (6.83%) 3: Unemployed: 362 (31.31%)	1: Employed/student: 1043 (90.22%) 2: On sick leave full-time: 57 (4.93%) 3: Unemployed: 56 (4.84%)
Baseline PA (0–10, worst to best)	9: 422 (5.55%) 8: 267 (3.51%) 7: 628 (8.26%) 6: 1237 (16.27%) 5: 1694 (22.28%) 4: 1162 (15.28%) 3: 1484 (19.52%) 2: 481 (6.33%) 1: 194 (2.55%) 0: 34 (0.45%)	6: 279 (24.13%) 4: 251 (21.71%) 7: 185 (16.00%) 5: 158 (13.67%) 8: 106 (9.17%) 3: 73 (6.31%) 10: 47 (4.07%) 2: 21 (1.82%) 9: 21 (1.82%) 1: 15 (1.30%)	6: 278 (24.05%) 7: 213 (18.43%) 4: 209 (18.08%) 8: 131 (11.33%) 5: 129 (11.16%) 3: 89 (7.70%) 10: 55 (4.76%) 9: 21 (1.82%) 2: 16 (1.38%) 1: 15 (1.30%)
Baseline pain (0–10, best to worst)	Min: 0 Max: 100 Mean: 47.21 SD: 22.31	Min: 0 Max: 100 Mean: 50.36 SD: 25.19	Min: 0 Max: 100 Mean: 47.48 SD: 25.45
Baseline QOL (0–10, worst to best)	Min: 0 Max: 100 Mean: 45.05 SD: 15.09	Min: 0 Max: 100 Mean: 51.83 SD: 19.19	Min: 0 Max: 100 Mean: 44.58 SD: 20.32
**Outcome**			
Changes in PA	Increased: 3131 (41.17%) Maintained: 2359 (31.02%) Decreased: 2113 (27.80%)	Increased: 405 (35.03%) Maintained: 354 (30.62%) Decreased: 397 (34.34%)	Increased: 438 (37.89%) Maintained: 349 (30.19%) Decreased: 369 (31.92%)
Changes in pain	Increased 20+ mm on the VAS: 597 (7.85%) Maintained: 4014 (52.78%) Decreased 20+ mm on the VAS: 2992 (39.37%)	Increased 20+ mm on the VAS: 239 (20.67%) Maintained: 586 (50.69%) Decreased 20+ mm on the VAS: 331 (28.63%)	Increased 20+ mm on the VAS: 265 (22.92%) Maintained: 579 (50.09%) Decreased 20+ mm on the VAS: 312 (26.99%)
Changes in QOL	Increased 10+ mm on the KOOS scale: 3687 (48.49%) Maintained: 3003 (39.49%) Decreased 10+ mm on the KOOS scale: 913 (12.01%)	Increased 10+ mm on the KOOS scale: 408 (35.29%) Maintained: 524 (45.33%) Decreased 10+ mm on the KOOS scale: 224 (19.38%)	Increased 10+ mm on the KOOS scale: 456 (39.45%) Maintained: 540 (46.71%) Decreased 10+ mm on the KOOS scale: 160 (13.84%)

## Data Availability

All the data for this study were acquired following a request to the GLA:D^®^ registry and Osteoarthritis Initiative, details of which can be found on their official websites: https://www.glaid.dk/index.html (accessed on 1 February 2023); https://nda.nih.gov/oai (accessed on 1 March 2024).

## Supplementary Materials

The following supporting information can be downloaded at: https://www.mdpi.com/article/10.3390/jcm14103406/s1. References [11,58,59,60] are cited in the Supplementary Materials file.

## Abbreviations

PA	Physical activity
QOL	Quality of life
OA	Osteoarthritis
GLA:D^®^	Good Life with osteoArthritis in Denmark
OAI	Osteoarthritis Initiative
RCTs	Randomized controlled trials
