# Supplementary material for: Are Changes in Physical Activity, Pain, and Quality of Life in Patients with Knee Osteoarthritis After Exercise Therapy and Education Beyond Normal Fluctuations? A Comparative Study
Source: J Clin Med. 2025 May 13;14(10):3406. doi: 10.3390/jcm14103406 (PMC12112693; doi:10.3390/jcm14103406)
Supplement: Supplementary file 1 [file jcm-14-03406-s001.zip › jcm-3582065-supplementary.pdf]

Supplementary Analysis

1. GLA:D® intervention

The GLA:D® program includes a standardized neuromuscular exercise component focused on improving strength, balance, and joint control. The 12 sessions consist of circuit-based group training that includes warm-up, core functional exercises targeting lower extremity and trunk muscles, and cool-down. Exercises are adapted in difficulty based on the participants' abilities and progression. Each session lasts approximately 60 minutes and is led by trained physiotherapists [11], [58], [59], [60].

**Component**	**Description**	**Duration per Session**	**Frequency**	**Total Duration**	**Who Delivers**
Educational sessions	Group-based education on osteoarthritis, self-management, and exercise benefits	60–90 minutes	2 sessions	2–3 weeks (before or alongside exercise)	GLA:D® Certified physiotherapist
Exercise Sessions	Circuit-based neuromuscular group training (strength, balance, joint control)	60 minutes	2x per week	6 weeks (12 sessions)	GLA:D® Certified physiotherapist
Exercise Structure	Includes warm-up, core functional exercises, and cool-down	Part of 60 60-minute session	2x per week	6 weeks (12 sessions)	GLA:D® Certified physiotherapist
Progression	Exercises are adapted in difficulty as the participant progresses	N/A	Ongoing	Throughout program	Physiotherapist
Assessment	- Functional tests (e.g., walking speed, chair stand) at baseline and follow-up<br><br>- PROMs	10–20 minutes	- At baseline & 3 months<br><br>- At baseline, 3, and 12 months	2 assessments	- Physiotherapist<br><br>- Online survey
Follow-up	Outcome tracking, advice, and support	Varies	- 3 months<br>- 12 months	Ongoing	Physiotherapist

			just PROMs online		Online survey

Table S1: Overview of the GLA:D® program components

2. Mapping

Because the UCLA scale used in the GLA:D® dataset and the PASE scale used in the OAI dataset are not the same—UCLA ranges from 1 to 10 (low to high) and PASE from 0 to 445—we needed to align them to ensure a valid comparison. To achieve this, we standardized the scoring scales by categorizing both into tertiles. The UCLA physical activity scores were divided into three categories: 1-4 (low), 5-6 (moderate), and 7-10 (high) based on established literature. Likewise, PASE scores were split into tertiles: Tertile 1 (low) from 0 to 152, Tertile 2 (moderate) from 153 to 207, and Tertile 3 (high) from 208 to 445, as guided by the relevant literature. This approach allowed us to directly compare physical activity (PA) levels between the two datasets, facilitating a more meaningful analysis of the effects of treatment programs on PA, quality of life (QOL), and pain intensity.

Figure S1 shows the distribution of PASE PA scores at baseline and after mapping based on the cut point. The left plot shows the original distribution of raw PASE scores, while the right plot displays the same scores categorized into tertiles (low, moderate, high) after standardization.

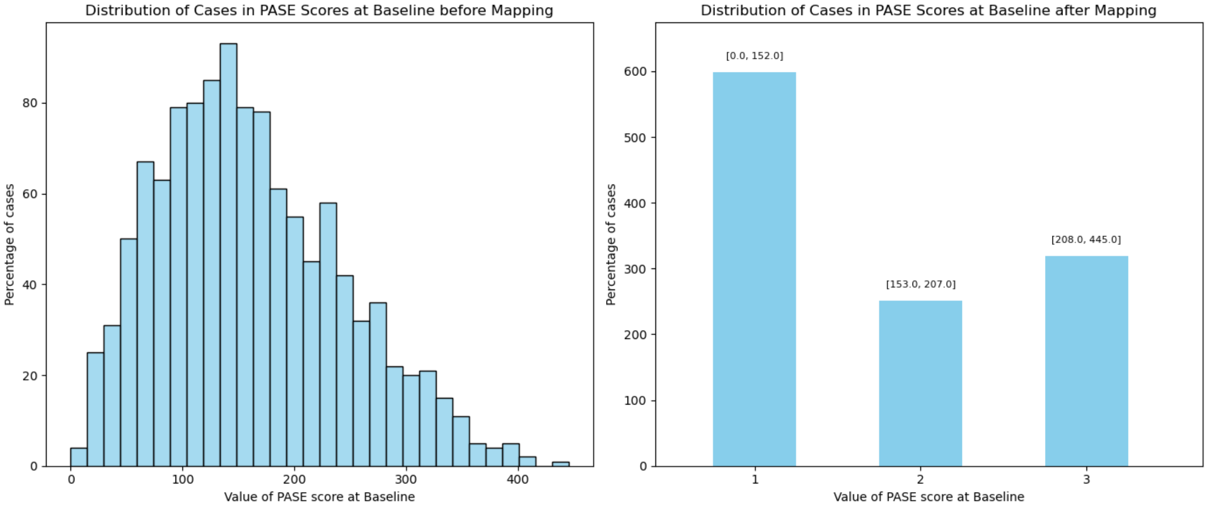

Figure S1: Distribution of PASE scores at baseline before and after Mapping. The left panel shows the continuous distribution of PASE scores, while the right panel presents the scores grouped into three categories: low (0–152), moderate (153–207), and high (208–445) for analysis.

In Figure S2, the distribution of UCLA PA scores at baseline is presented. The left plot shows the original UCLA score distribution, and the right plot illustrates the scores categorized into tertiles to match the standardized classification for comparison with PASE.

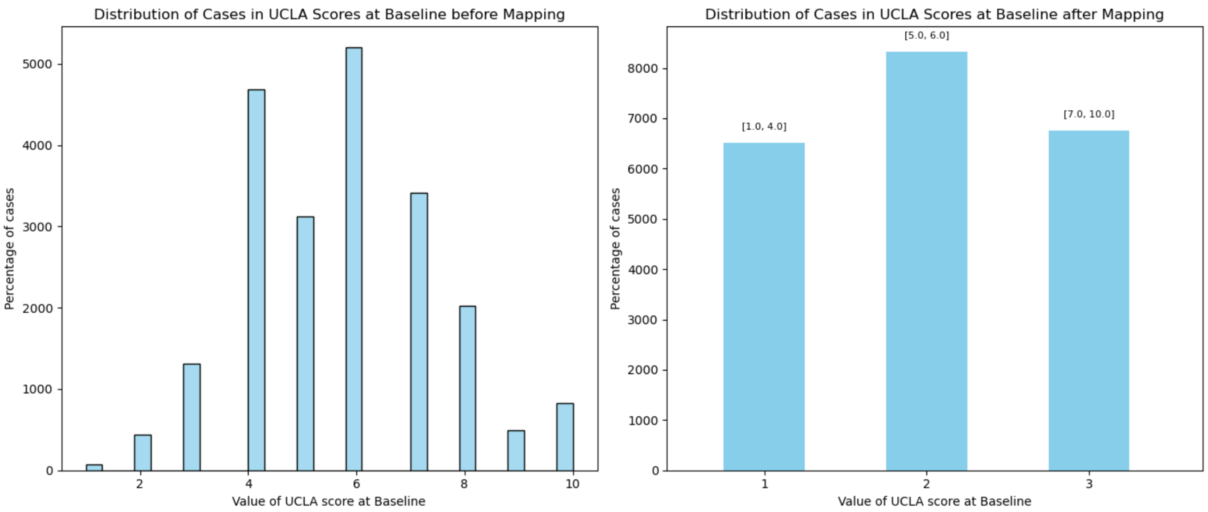

Figure S2: Distribution of UCLA scores at baseline before and after Mapping. The left panel shows the distribution of UCLA scores at baseline, while the right panel presents the scores grouped into three categories: low (1–4), moderate (5–6), and high (7–10) for analysis.

In Figure S3, the comparison of changes in PA between the treatment, control, and balanced control groups is shown. The bar chart illustrates the proportion of cases that experienced increased, maintained, or decreased PA across these groups.

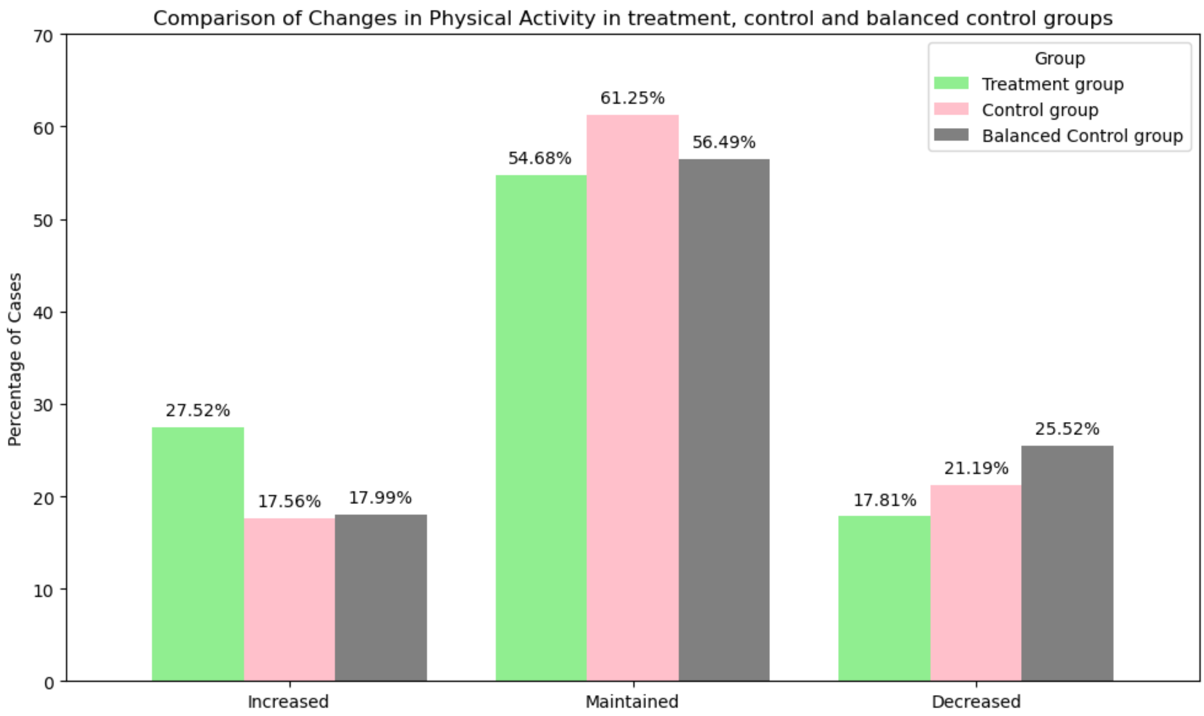

Figure S3: Changes in PA from baseline to follow-up in treatment, control, and balanced control groups

A Chi-squared test was performed to assess the association between the treatment and balanced control groups in terms of PA changes. The results yielded a Chi-squared value (Chi²) of 64.41, a p-value of 1.03e-14, and 2 degrees of freedom. The p-value (1.03e-14) is far below the 0.05 significance threshold, indicating a highly statistically significant association between

the treatment and balanced control groups. This strengthens the conclusion that the treatment program had a notable impact on PA levels.

3. Adding pain medication to the analysis:

In this analysis, we included pain medication as an additional confounding factor to assess its influence on the observed outcomes. The number of cases in the GLA:D® dataset remained unchanged, while in the OAI dataset, the number of cases slightly decreased from 1156 to 1154.

Including pain medication as a co-founding factor slightly shifts the results but does not alter the overall trends. The treatment group consistently shows the best outcomes in pain reduction, QOL, and PA. With pain medication, control groups show slight improvements in pain stability, while the treatment group sees minor decreases in significant improvements. However, the intervention remains more effective than medication alone, reinforcing its benefits.
The Chi-squared tests indicate significant differences between the treatment and balanced control groups across all outcome measures.

Pain reduction shows the strongest association with treatment (chi-squared value of 280.18, with a p-value of 1.43 and 2 degrees of freedom), suggesting a highly significant effect of the intervention.

QOL improvements also show a strong association (chi-squared value of 31.30, with a p-value of 1.59 and 2 degrees of freedom), reinforcing the intervention's impact.

PA changes are significant but weaker (chi-squared value of 8.45, with a p-value of 0.01 and 2 degrees of freedom), indicating a moderate association. Overall, these results confirm that the treatment group experiences significantly better outcomes compared to the balanced control group.

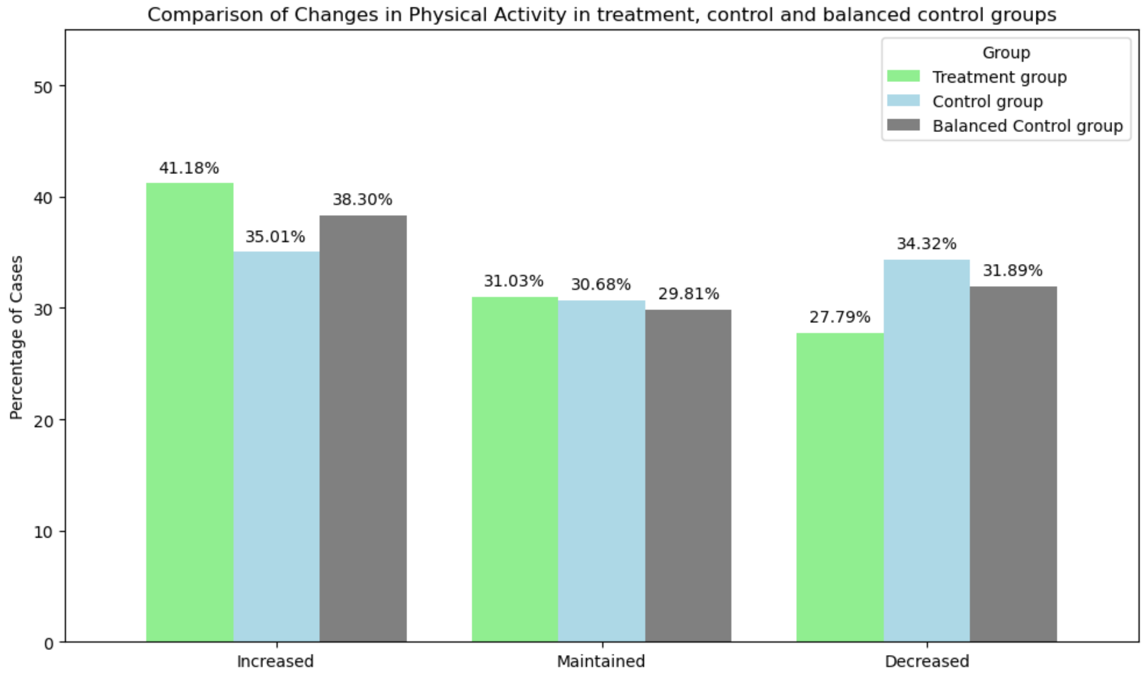

Figure S4. Changes in PA from baseline to follow-up in treatment, control, and balanced control groups with an additional co-founder "pain medication."

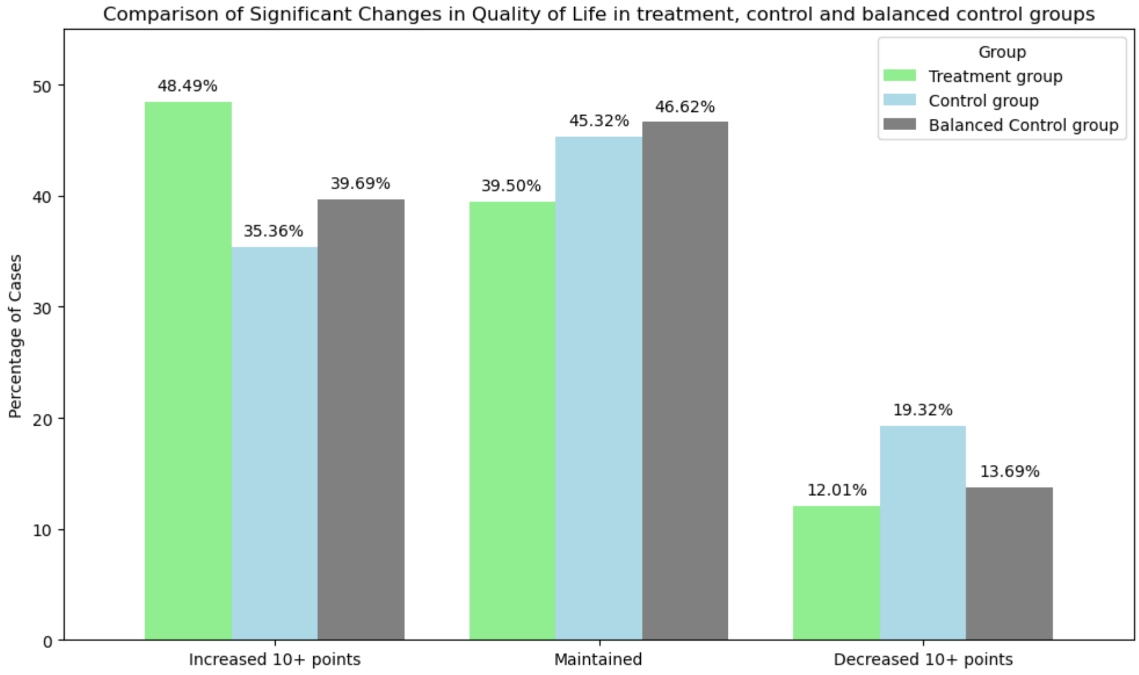

Figure S5. Changes in QOL from baseline to follow-up in treatment, control, and balanced control groups with an additional co-founder “pain medication.

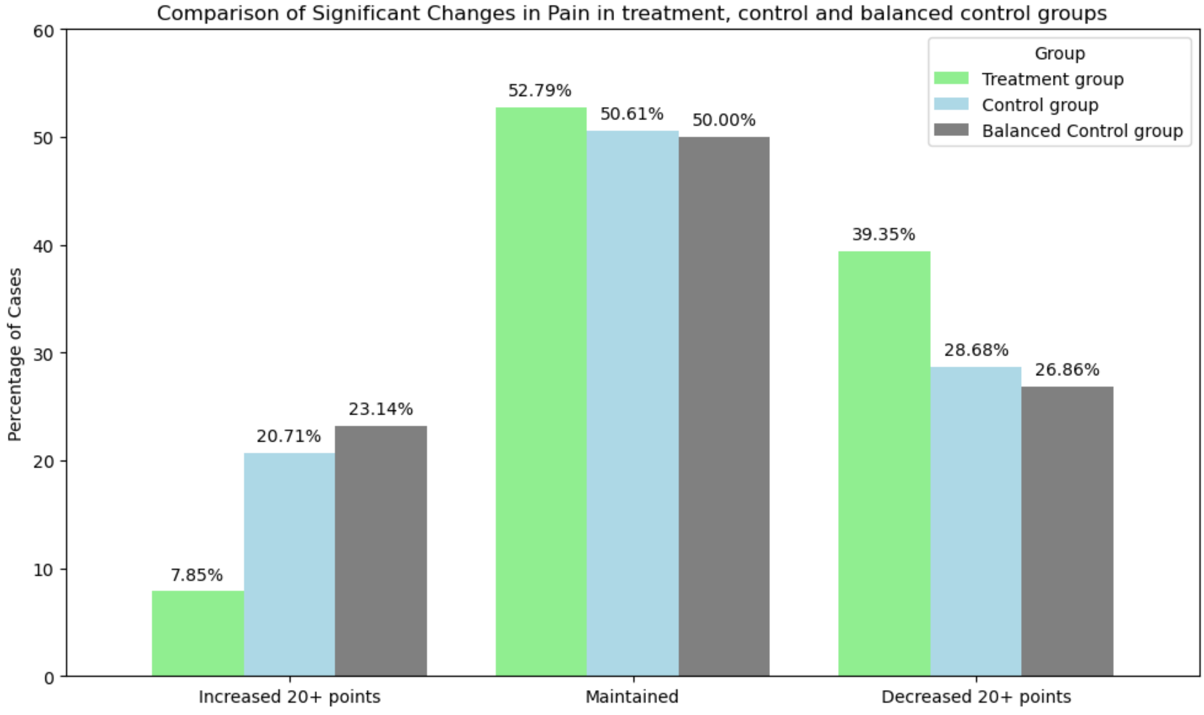

Figure S6. Changes in pain intensity from baseline to follow-up in treatment, control, and balanced control groups with an additional co-founder “pain medication.

3. Additional variable

GLA:D®				OAI			
**Physiotherapy**	Baseline	Has the patient previously consulted a physiotherapist because of the current joint problems?	0: 4,882 (64.21%)<br><br>1: 2,721 (35.79%)	**Physiotherapy**	Baseline: Enrollment	Currently seeing chiropractic care practitioner for arthritis or joint pain	NaN: 1,045 (90.40%)<br><br>0: 46 (3.98%)<br><br>1: 65 (5.62%)
	12 months follow-up	Since starting up GLAD, have you consulted a physiotherapist because of the hip/knee problems (other than those related to GLAD)?	NaN:7,603 (100%)		Follow-up visit Interview (12-month Annual)	Currently seeing chiropractic care practitioner for arthritis or joint pain	NaN:1,064 (92.04%)<br><br>0: 32 (2.77%)<br><br>1: 60 (5.19%)
**Pain medication**	Baseline	Does the patient take any pain medications, including herbal or dietary supplements? (Ask the patient to think about the last 2 weeks)	0: 1,957 (25.74%)<br><br>1: 5,646 (74.26%)	**Pain medication**	Baseline: Enrollment	Used nonprescription or prescription NSAIDS (e.g., Aspirin, Ibuprofen…) for joint pain or arthritis more than half the days of the month, past 30 days	NaN: 2 (0.17%)<br><br>0: 786 (67.99%)<br><br>1: 368 (31.83%)
	12 months follow-up	Do you take any pain medications (except herbal or dietary supplements) because of your knee or hip?	NaN:2,971 (39.08%)<br><br>0: 2,553 (33.58%)<br><br>1: 2,079 (27.34%)		Follow-up visit Interview (12-month Annual)	Used nonprescription or prescription NSAIDS (e.g., Aspirin, Ibuprofen…) for joint pain or arthritis more than half the days of the month, past 30 days	NaN: 255 (22.06%)<br><br>0: 651 (56.31%)<br><br>1: 250 (21.63%)
**Knee replacement**	Baseline	Have you had a joint	NaN: 52 (0.68%)	**Knee replacement**	Baseline: Enrollme	Left or right knee, ever had	NaN: 98 (8.48%)

		replacement in a hip or knee?	0: 6,789 (89.29%)<br><br>1: 762 (10.02%)		nt	knee replacement surgery	0: 1,042 (90.14%)<br><br>1: 16 (1.38%)
	12 months follow-up	Have you had a knee or hip replacement since starting up in GLA:D?	NaN:2,790 (36.70%)<br><br>0: 4,449 (58.52%)<br><br>1.0: 364 (4.79%)		Follow-up visit Interview (12-month Annual)	Left or right knee, ever had knee replacement surgery	NaN:1053 (91.09%)<br><br>0: 100 (8.65%)<br><br>1: 3 (0.026%)

Table S2. Characteristics of the additional variables respected in the supplementary analyses of all included participants. A value of 0 indicates "No," 1 indicates "Yes," and NaN represents a missing value.

References:

[11] S. T. Skou and E. M. Roos, "Good Life with osteoArthritis in Denmark (GLA:D™): evidence-based education and supervised neuromuscular exercise delivered by certified physiotherapists nationwide," *BMC Musculoskelet. Disord.*, vol. 18, no. 1, p. 72, Feb. 2017, doi: 10.1186/s12891-017-1439-y.

[58] "GLA:D® International Network," Glad International. Accessed: Apr. 16, 2025. [Online]. Available: https://gladinternational.org/

[59] "Structured Education and Neuromuscular Exercise Program for Hip and/or Knee Osteoarthritis: A Health Technology Assessment," *Ont. Health Technol. Assess. Ser.*, vol. 18, no. 8, pp. 1–110, Nov. 2018.

[60] "GLA:D Physiotherapy Program for Osteoarthritis Relief – Proven Approach - Exercise Classes, Physiotherapist Supervised," Thrive Physio Plus. Accessed: Apr. 16, 2025. [Online]. Available: https://www.thrivephysioplus.com.au/services/glad-physiotherapy/
